# Unusual Neurologic Manifestations of a Patient with Cyanotic Congenital Heart Disease after Phlebotomy

**DOI:** 10.1155/2015/924862

**Published:** 2015-04-02

**Authors:** Hooman Salimipour, Somayeh Mehdizadeh, Reza Nemati, Mohamad Reza Pourbehi, Gholam Reza Pourbehi, Majid Assadi

**Affiliations:** ^1^Department of Neurology, Bushehr University of Medical Sciences, Bushehr 7514633341, Iran; ^2^The Persian Gulf Tropical Medicine Research Center, Bushehr University of Medical Sciences, Bushehr 7514763448, Iran; ^3^Department of Cardiology, Bushehr University of Medical Sciences, Bushehr 7514633341, Iran; ^4^The Persian Gulf Nuclear Medicine Research Center, Bushehr University of Medical Sciences, Bushehr 7514633341, Iran

## Abstract

Secondary erythrocytosis in cyanotic congenital heart disease (CCHD) is a compensatory response to chronic hypoxia which should be managed with caution. CCHD patients, who have compensated erythrocytosis but do not manifest significant neurologic symptoms, may experience secondary life-threatening complications such as stroke in case of inappropriate phlebotomy. This study reports a young man with CCHD who developed frequently repeated transient neurologic deficits with various presentations after one session of phlebotomy. The symptoms resolved a few days after the hematocrit (Hct) level returned to the prephlebotomy level.

## 1. Introduction

Secondary erythrocytosis is a physiological response to tissue hypoxia in which there are an increased number of circulating red blood cells, an improved oxygen carrying capacity, and an enhanced blood viscosity. This adaptation results in sufficient tissue oxygenation at a higher blood viscosity and Hct; however this equilibrium may be impaired if excessive erythrocytosis impairs oxygen delivery by increasing blood viscosity and eventually inverses the beneficial effects of erythrocytosis [1]. Cerebrovascular event has been previously considered as one of the most serious complications of secondary erythrocytosis in CCHD, although a large study in adult patients with CCHD showed no increased risk of stroke [2].

Patients with secondary erythrocytosis related to CCHD can be divided into two groups as follows: first group with stable “compensated” erythrocytosis, adequate iron stores, and absent or mild hyperviscosity symptoms and second group with “decompensated” erythrocytosis, iron deficiency states, and recurrent hyperviscosity symptoms [3].

For patients with compensated secondary erythrocytosis, phlebotomy did not indicate any usefulness when they exhibit mild, moderate, or no neurologic symptoms related to cerebral hyperviscosity. Repeated phlebotomy was associated with iron stores depletion, microcytosis, blood hyperviscosity, and impaired oxygen delivery [4].

In this paper, we report a 35-year-old man with complex CCHD, who showed unusual presentation of recurrent various transient neurologic deficits after first therapeutic phlebotomy.

## 2. Case

A 35-year-old man is presented here as a known case of complex CCHD including dextrocardia, ventricular septal defect, transposition of the great arteries, atrial septal defect, pulmonary stenosis, with a history of previous admission for cardiac evaluation, and catheterization for diagnosis of heart anatomy and RV pressure 25 years ago. However, the patient has not shown any symptoms and no surgical intervention has been done on the patient. He had been feeling well until 2 days before admission when he reported some neurological symptoms like dizziness, tingling, and fatigue. He was visited by an internist who advised him to undergo a phlebotomy, 250 cc with normal saline replacement, for three continuous days due to his high Hct level (Hct: 64.5). After the first session, Hct and hemoglobulin (Hb) oxygen saturation were 58 and 76%, respectively. At the second session of phlebotomy, immediately after several unsuccessful painful intravenous angiocut catheter insertions, the patient developed tongue and right body tingling and paresthesia, later progressed to dysarthria and stiffness and tightness in right extremities which were resolved in about 20 seconds. During recovery, the same symptoms occurred in the left upper limb which were all resolved completely in about 30 seconds. Emergency brain CT has not showed cerebral hemorrhage or infarction and brain magnetic resonance imaging (MRI) and diffusion weighted MR has not revealed any more information. Transthoracic echocardiography did not also show obvious thrombus. The patient was admitted in stroke unit and hydration, medication with aspirin 80 mg, and infusion of heparin with rate of 1000 unit per hour started. Although the patient was on antiplatelet and anticoagulant, his symptoms frequently recurred in various forms alternating in both sides: right and left hemiparesis, stiffness, loss of dexterity, chorea and dystonic movement, facial and tongue tingling, and paresthesia. During symptomatic period, neurologic examination revealed transient Babinski signs at several times. The patient's complaints continued for several days and since day 4 after presentation, frequency and severity of symptoms decreased. Brain and neck MR angiography, cervical MRI, Transcranial Doppler (TCD) sonography and monitoring, and electroencphalography were normal. Other blood count parameters (white blood cell: 7000 cells/mcL, mean corpuscular volume: 84 fL, and platelet count: 155000 cells/mcL), serum iron (88 *μ*g/dL), ferritin (110 ng/mL), and homocysteine (13 *μ*mol/L) were in normal range. After discharge of the patient from hospital, frequency and severity of the neurologic attacks gradually decreased coinciding with an increase in Hb and Hct. Terminally, his complaint resolved at an Hct level (62.7%) and an Hb O_2_ saturation (85%) near to the amount before admission. One month later, the patient only occasionally complained from a limb tingling similar to the symptom before phlebotomy.

## 3. Discussion

Our patient manifested repeated transient neurologic deficits after the first session of phlebotomy and his symptoms were compatible with the involvement of various cerebral areas, probably originating from an ischemic process.

Polycythemic cyanotic patients experience two different symptoms of hyperviscosity symptoms and thrombotic event [1]. The former symptom, hyperviscosity, can sometimes be misinterpreted as thrombotic event and lead to inappropriate phlebotomies [3]. Hyperviscosity is generally associated with impaired tissue perfusion and tissue oxygen delivery which causes symptoms such as headache, visual disturbance, loss of concentration, paresthesia, muscle weakness, and fatigue [3].

The more serious presentation of polycythemia is thrombotic event inclusive of cerebrovascular accident (CVA) and transient ischemic attacks (TIA) which are usually manifested [3] as symptoms or signs of hemiparesis and/or hemisensory defects, cortical blindness, aphasia, dysarthria, dysphagia ataxia, or drop attacks, distinguishing thrombotic event from hyperviscosity [5].

Our patient had both types of symptoms: firstly he presented limb tingling and dizziness that was related to hyperviscosity, but after a single therapeutic phlebotomy, he developed unusual various transient symptoms that seemed to have resulted from ischemia. Several mechanisms might be involved in cerebral ischemia in patients with CCHD like hyperviscosity, iron deficiency anemia, thrombosis, or associated vascular risk factor [2, 3].

Despite a correlation between Hct level and vascular occlusive in polycythemia vera (PV), the association between hyperviscosity and vascular thrombosis is not yet reported in adult patients with CCHD and secondary erythrocytosis [3]. Although PV is associated with vascular accident such as stroke [5], Perloff et al. did not report an increased incidence of cerebral infarction in adult patients who suffer from CCHD and secondary erythrocytosis [4]. Ammash and Warnes reported an increased risk of CVAs in adult patients with CCHD which related to hypertension, atrial fibrillation, repeated phlebotomies, and iron deficiency, but they have not mentioned any correlation between Hg or Hct level and stroke [2].

Therapeutic guidelines in PV recommend maintaining Hct level lower than 45 through phlebotomy. However, it is not proved that phlebotomy is associated with a lower risk of thrombosis, because iron deficiency resulting from frequent phlebotomy may even cause a higher incidence of vascular occlusion [1].

Patients with CCHD who do not have hyperviscosity symptoms are not advised to have phlebotomy unless patient is symptomatic at Hct level above 65 and is not dehydrated [1]. Dehydration and iron deficiency may precipitate or aggravate symptomatic hyperviscosity and thrombotic event and have to be managed before phlebotomy in patients with CCHD with Hct level of >65% [3].

If venesection is performed without volume replacement in patient suffering from dehydration, the consequences of the acute fall in systemic blood flow and oxygen delivery can impair cardiovascular and cerebral perfusion [1]. Iron deficiency anemia is associated with microcytic erythrocytes that are rigid and difficult to pass through microcirculation, ultimately increasing the risk of hyperviscosity symptoms and CVAs in patients with secondary erythrocytosis [3].

Our patient did not have iron deficiency because one session of phlebotomy is not enough for iron depletion to occur and serum iron and ferritin were also in a normal range. Suspicious of probable dehydration, the patient was treated with sufficient intravenous solution, which did not resolve the patient's symptoms.

Dinia et al. reported a 29-year-old woman with a corrected CCHD suffering from advancing pulmonary hypertension and secondary erythrocytosis (Hct: 63%). After two sessions of therapeutic phlebotomy, the patient experienced three instances of TIA that disappeared immediately after the infusion of heparin. The Hct value after first phlebotomy was 57.5%. Bilateral TCD monitoring showed several microembolic signals (MES) on middle cerebral arteries of both sides which disappeared after anticoagulant therapy. The authors believe that the clinical events may be due to hyperviscosity and microembolization of aggregated red blood cells [6]. However, in our patient, treatment with heparin infusion did not resolve neurologic episodes which were frequently repeated every few minutes. TCD sonography and monitoring have not shown any significant arterial stenosis and MES, respectively. MES considered silent cerebral emboli that are usually detected during TCD monitoring of the intracranial arteries [7]. There seems to be another mechanism involved in pathogenesis of neurologic episodes in our case.

Despite improvement of tissue oxygen delivery and cerebral blood flow following phlebotomy, blood oxygen carrying capacity decreases in secondary erythrocytosis [8].

Our case had an Hct level about 64.5% and an Hb O_2_ saturation around 85%, which decreased after phlebotomy to 58% and 76%, respectively. His neurologic deficit attacks appeared after a decrease in Hct levels and Hb oxygen saturation. Gradually, there was a cut in the frequency of these attacks and their eventual disappearance coincided with an increase in Hb O_2_ saturation and Hct level to the prephlebotomy status. It is possible that phlebotomy has caused a reduced arterial oxygen saturation and finally might be manifested as neurologic deficits.

In conclusion, our case manifested unusual neurologic deficits after one session of inappropriate phlebotomy. The deficits resolved only after Hct level reached the prephlebotomy level. It is important to note that lack of knowledge about the abovementioned unusual presentations as a result of injudicious phlebotomy and accompanying probable mechanisms may result in inappropriate and occasionally life-threatening treatments such as anticoagulation therapy.

## References

[1] Thorne S. A. (1998). Management of polycythaemia in adults with cyanotic congenital heart disease. *Heart*.

[2] Ammash N., Warnes C. A. (1996). Cerebrovascular events in adult patients with cyanotic congenital heart disease. *Journal of the American College of Cardiology*.

[3] Rose S. S., Shah A. A., Hoover D. R., Saidi P. (2007). Cyanotic congenital heart disease (CCHD) with symptomatic erythrocytosis. *Journal of General Internal Medicine*.

[4] Perloff J. K., Marelli A. J., Miner P. D. (1993). Risk of stroke in adults with cyanotic congenital heart disease. *Circulation*.

[5] Costa S., Marques J., Barradas A., Valverde A. (2011). Transient spinal cord ischemia as presenting manifestation of polycythemia vera. *Case Reports in Neurology*.

[6] Dinia L., Rizzi D., Gandolfo C., del Sette M. (2006). Disappearance of microembolic signals after heparin in acute cerebral ischemia due to cyanotic heart disease with polyglobulia. *Cerebrovascular Diseases*.

[7] Shariat A., Yaghoubi E., Nemati R., Moaref A. R., Aghasadeghi K., Haghighi A. B. (2012). Microembolic signals in patients with cryptogenic stroke with or without patent foramen ovale. *Journal of Stroke and Cerebrovascular Diseases*.

[8] DeFilippis A. P., Law K., Curtin S., Eckman J. R. (2007). Blood is thicker than water: the management of hyperviscosity in adults with cyanotic heart disease. *Cardiology in Review*.

